# WNT3A Promotes Neuronal Regeneration upon Traumatic Brain Injury

**DOI:** 10.3390/ijms21041463

**Published:** 2020-02-21

**Authors:** Chu-Yuan Chang, Min-Zong Liang, Ching-Chih Wu, Pei-Yuan Huang, Hong-I Chen, Shaw-Fang Yet, Jin-Wu Tsai, Cheng-Fu Kao, Linyi Chen

**Affiliations:** 1 Institute of Molecular Medicine, National Tsing Hua University, Hsinchu 30013, Taiwan; chyju13180@hotmail.com (C.-Y.C.); liang4646@gmail.com (M.-Z.L.); shps982104@gmail.com (P.-Y.H.); red81820@gmail.com (H.-I.C.); 2 Department of Life Science, National Tsing Hua University, Hsinchu 30013, Taiwan; liz110645@gmail.com; 3 Institute of Cellular and System Medicine, National Health Research Institutes, Zhunan 35053, Taiwan; shawfang@icloud.com; 4 Institute of Brain Science, National Yang-Ming University, Taipei 11221, Taiwan; tsaijw@ym.edu.tw; 5 Institute of Cellular and Organismic Biology, Academia Sinica, Taipei 11574, Taiwan; 6 Department of Medical Science, National Tsing Hua University, Hsinchu 30013, Taiwan

**Keywords:** neuronal regeneration, traumatic brain injury, WNT3A, cortical neurons

## Abstract

The treatment of traumatic brain injury (TBI) remains a challenge due to limited knowledge about the mechanisms underlying neuronal regeneration. This current study compared the expression of *WNT* genes during regeneration of injured cortical neurons. Recombinant WNT3A showed positive effect in promoting neuronal regeneration via in vitro, ex vivo, and in vivo TBI models. Intranasal administration of WNT3A protein to TBI mice increased the number of NeuN^+^ neurons without affecting GFAP^+^ glial cells, compared to control mice, as well as retained motor function based on functional behavior analysis. Our findings demonstrated that WNT3A, 8A, 9B, and 10A promote regeneration of injured cortical neurons. Among these WNTs, WNT3A showed the most promising regenerative potential in vivo, ex vivo, and in vitro.

## 1. Introduction

Traumatic brain injury (TBI) is a major cause of morbidity and lifelong disability, making it a critical healthcare concern worldwide. Globally, an estimated 69 million individuals per year suffer from TBI, and according to the Centers for Disease Control and Prevention, it accounts for over 30% of injury-related deaths in the United States [1,2,3]. There is currently no effective treatment that can stimulate regeneration, partly due to the heterogeneity of pathophysiology, severity, and outcomes, so much of the damage/dysfunction in TBI patients becomes permanent [4]. Among over 200 registered ongoing or completed clinical trials for TBI, administration of chemical compounds (e.g., sertraline, amantadine, minocycline), transplantation of autologous bone marrow-derived cells, and other interventions may partially rehabilitate impaired consciousness and/or improve neurocognitive dysfunction. Nevertheless, a large number of these trials have been terminated (ClinicalTrials.gov). So, the medical burden of TBI highlights the pressing need to develop better treatments for brain injury.

WNT signaling is an evolutionarily conserved pathway that regulates axis specification of the neural plate, neural tube morphogenesis, dendrite and axon development, and synaptic plasticity [5,6,7]. Aberrant WNT signaling has previously been implicated in Alzheimer’s disease and Parkinson’s disease [8,9,10]. In this study, we determined the expression of a number of *WNT* genes during regeneration of injured cortical neurons. In vitro and in vivo studies examined the regenerative potential of WNT3A upon traumatic brain injury.

## 2. Results

### 2.1. Identification of WNT Genes as RAGs for Axonal Regeneration of Cortical Neurons

**Figure 1:**
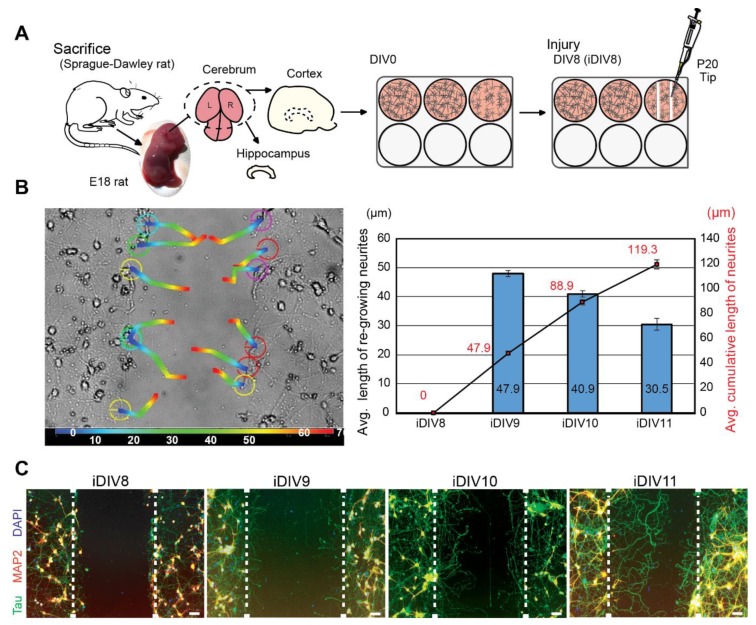
Neurite re-growth of primary cortical neurons in an in vitro traumatic brain injury (TBI) model. (**A**) Schematic flow chart of experimental design. (**B**) Trajectory of single neurite re-growth was tracked from 0 h (blue) to 72 h (red) after injury using tracking function of AxioVision software (left). Quantitative results of average length of re-growing neurite (blue bars) and average cumulative length (line graph) are shown from iDIV8 to iDIV11 (right). (*n* = 3, track 12 neurites/experiment.) (**C**) Cortical neurons were fixed on iDIV8 to iDIV11 and were subjected to immunofluorescence staining with anti-Tau (green, axonal marker), anti-MAP2 (red, dendritic marker) antibodies, and DAPI (blue, nuclei). Merged images are shown. White dashed lines indicate the borders of the injured gap. Scale bar, 50 µm.

To evaluate neuronal regeneration, rat cortical neurons were cultured in vitro. On DIV (day in vitro) 8, primary cortical neurons were injured by scraping lines through the culture with a p20 tip (Figure 1A). It was estimated that 10% neurons were injured via this approach. An anti-mitotic reagent, AraC, was added to the culture medium to inhibit proliferation of glial cells [11,12]. Regenerating neurites were tracked for 72 h after injury. Regeneration of injured cortical neurons was most prominent within 48 h (injured DIV10, iDIV10) after the injury (Figure 1B). Immunofluorescence staining using anti-microtubule-associated protein 2 (MAP2, marker for dendrites) and anti-Tau (axonal marker) antibodies demonstrated that the re-growing neurites were mostly Tau^+^ axons (Figure 1C).

**Figure 2:**
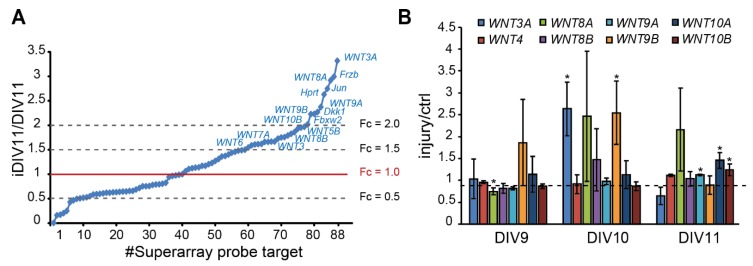
Up-regulation of *WNT* genes during regeneration of injured cortical neurons. (**A**) Differential expression profile of WNT-related genes by superarray assay is shown. Genes with an expression fold-change above 1.5 in response to injury are denoted. (**B**) Analysis of differential expression of *WNT*s by qPCR during neuronal regeneration. Data are presented as mean ± SEM from at least three independent experiments (*WNT3A*, *8A*: *n* = 4; *WNT9B*, *10A*: *n* = 5). * *p* ≤ 0.05 (paired Student’s *t*-test).

To identify candidate regeneration-associated genes (RAGs) during regeneration of injured cortical neurons, our previous RNA-seq data suggest a subset of *WNT* genes as candidate RAGs (data not shown), in addition to pathway-focused superarray analysis of 88 *WNTs* and WNT target genes, given that our previous data has suggested that WNT signaling is implicated in neuronal regeneration (data not shown). Superarray analysis showed that 48 genes (54.5%) were up-regulated. Among them, *WNT3*, *WNT3A, WNT4, WNT5A, WNT5B, WNT6, WNT7A, WNT8A, WNT8B, WNT9A, WNT9B,* and *WNT10B* expression were up-regulated (fold change > 1.5) during regeneration (Figure 2A). Increased expression of *WNT3A, WNT8A,* and *WNT9B* on iDIV10 compared to DIV10 was validated by qPCR, with the expression of *WNT3A* and *WNT9B* showing significant increase (Figure 2B). Although the fold increase of *WNT* genes is not high, local concentration of secreted WNTs in the brain tissue under tight control of blood–brain barrier (BBB) may be sufficient to trigger cellular response.

### 2.2. WNT3A Recombinant Protein Promotes Neuronal Regeneration and Functional Recovery

**Figure 3:**
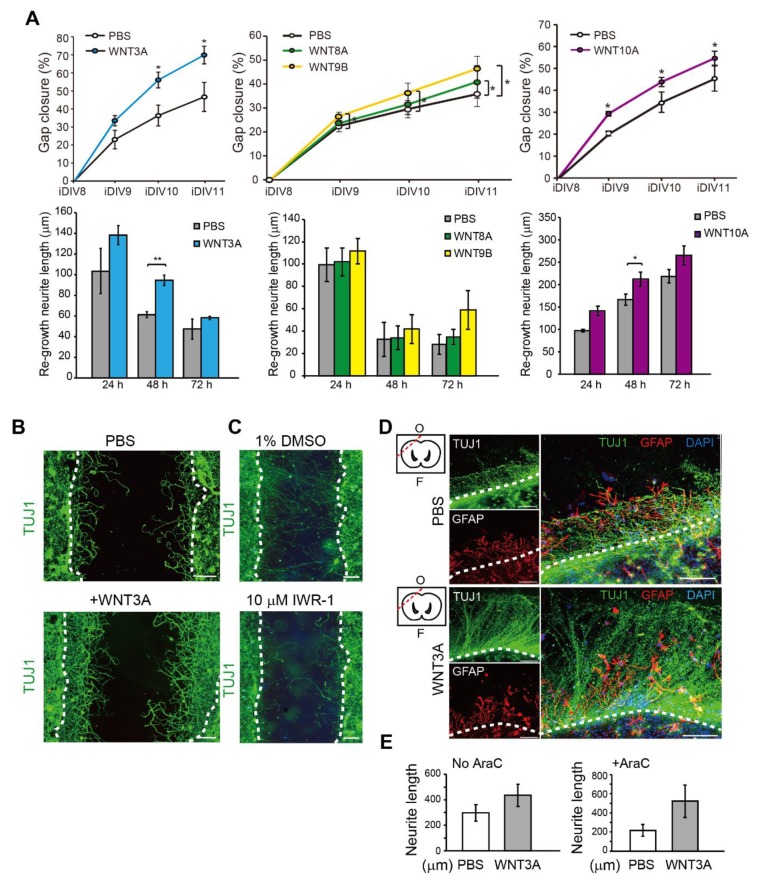
Recombinant WNT3A promotes regeneration of injured cortical neurons and brain tissues. (**A**) Cortical neurons were pre-treated with PBS, WNT3A, WNT8A, WNT9B, or WNT10A recombinant proteins prior to injury on DIV8. Upper: The percentages of gap closure were calculated from iDIV8 to iDIV11. Bottom: The lengths of re-growing neurites were measured between 0–24 h, 24–48 h, and 48–72 h after injury. Data are presented as mean ± SEM (*n* = 4 for WNT3A and WNT9B experiments; *n* = 5 for WNT10A experiment). * *p* ≤ 0.05, ** *p* ≤ 0.01 (paired Student’s *t*-test). (**B**) Cortical neurons with or without WNT3A (50 ng/mL) pre-treatment were fixed and subjected to immunofluorescence staining with anti-TUJ1 antibody (neurites) on iDIV11. (**C**) Cortical neurons were treated with 1% DMSO or IWR-1 prior to injury on DIV8. Neurons were subjected to immunofluorescence staining with anti-TUJ1 antibody on DIV11 and the representative images are shown. Dashed lines in (**B**,**C**) indicate the borders of the injured gap. Scale bar, 100 µm. (**D**) The effect of WNT3A on neuronal regeneration was assessed using organotypic brain slice culture. Brain slices were injured at the olfactory tubercle on DIV0, as indicated by the red dashed line in the upper left atlas map, and cultured with or without WNT3A (50 ng/mL) in AraC-containing medium. Brain tissues were fixed on iDIV4 and subjected to immunofluorescence staining with anti-TUJ1 (green) and anti-GFAP (red, glia) antibodies, and DAPI (blue). The border of injured sites is depicted by white dashed lines, and remaining brain tissue is underneath the lines in each panel. F: Frontal lobe; O: Occipital lobe. Scale bar, 100 µm. (**E**) The length of regenerating neurites from the brain slices treated with PBS, WNT3A, AraC + PBS, or AraC + WNT3A were calculated. Data are presented as mean ± SEM (*n* = 4). Data were analyzed using non-parametric Mann–Whitney test.

To address the effect of WNT proteins on the regeneration of injured cortical neurons directly, WNT3A, WNT8A, WNT9B, and WNT10A recombinant proteins were added. The percentage of relative regeneration was determined as average distance of neurite re-growth compared to the initial width of the injury gap. The lengths of re-growing neurites during 0–24 h, 24–48 h, and 48–72 h after neuronal injury were shown. As shown in Figure 3A,B, the regeneration of injured cortical neurons was improved by 20–25% in the presence of WNT3A compared to without WNT3A by 72 h (iDIV11). The addition of WNT8A, WNT9B, and WNT10A also enhanced regeneration of injured cortical neurons, though not to the same extent as WNT3A did (Figure 3A).

**Figure 4:**
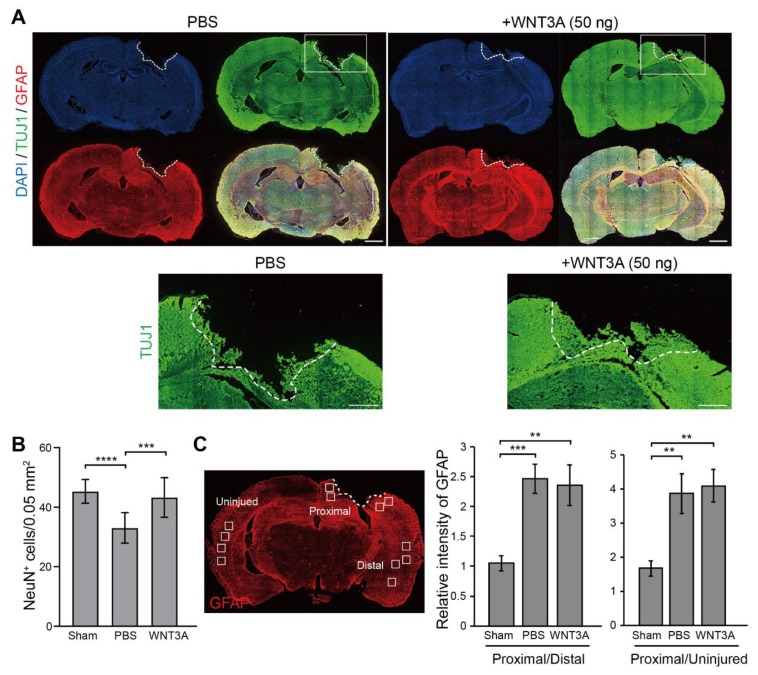
Recombinant WNT3A promotes regeneration of injured brain tissue of controlled cortical impact (CCI) mice. (**A**) The effect of WNT3A on neuronal regeneration was assessed using in vivo CCI mouse model. Cortical impact on motor and somatosensory cortex was generated and the mice were given PBS or 50 ng WNT3A intranasally during 0–3 dpi. Injured brain tissues were fixed, cryosectioned on 4 dpi, and subjected to immunofluorescence staining with anti-TUJ1 (green), anti-GFAP (red) antibodies, and DAPI (blue). White dashed lines depict the border of CCI sites. The regions of interest (ROIs) are indicated in the boxed regions. Scale bar, 1 mm. Enlarged images of the ROIs are shown at the bottom. Scale bar, 0.5 mm. (**B**) The number of NeuN^+^ cells and (**C**) the relative intensity of GFAP of CCI cryosections from sham group (*n* = 10), PBS-treated (*n* = 12) and WNT3A-treated (*n* = 8) CCI animals were quantified as described in the Materials and Methods. GFAP-immunostained cryosection on the left panel in (**C**) defines 0.05 mm^2^-ROIs within white boxes. Dashed lines indicate the borders of the impact site. Data are presented as mean ± SEM. ** *p* ≤ 0.01, *** *p* ≤ 0.001, **** *p* ≤ 0.0001, analyzed by one-way ANOVA followed by Tukey’s test.

Thus, the WNT3A-dependent regeneration was further examined by treating injured cortical neurons with IWR-1, a WNT3A inhibitor, and neuronal regeneration was inhibited (Figure 3C). To assess the effect of WNT3A in a multicellular three-dimensional (3D) culture, organotypic brain slice culture was used. Brain slices were injured in the olfactory tubercle, followed by WNT3A or PBS treatment (Figure 3D). In order to reduce injury-induced glial cell proliferation, which has been shown to hinder neurite re-growth, the effect of combining WNT3A + AraC or PBS + AraC was also compared. Injured brain slice treated with WNT3A increased the length of regenerating neurites compared to PBS control (Figure 3D,E). To show the in vivo effect of WNT3A administration on neuronal regeneration, we adapted one of the TBI models, controlled cortical impact (CCI) injury mice model. CCI model mimics post-TBI neuro-pathophysiology, resembling cognitive or behavioral deficits seen in human TBI patients [13,14,15,16]. C57BL/6J mice were injured via CCI at 3 m/s in speed and 1 mm in depth to generate mild TBI, followed by daily intranasal administration of 50 ng WNT3A recombinant protein for consecutive four days. As demonstrated in Figure 4A, administration of WNT3A robustly enhanced regeneration of brain tissue on 4 dpi (day post injury), characterized by better healing of the injured area compared to vehicle treatment. Within the regenerated tissue, there were increased NeuN^+^ (neuronal marker) cells for WNT3A-treated mice compared to PBS-treated control group (Figure 4B). Upon injury, increased glial fibrillary acidic protein (GFAP) reflects injury-induced proliferation of glial cells. GFAP^+^ signal was compared between proximal regions to the injury site, ipsilateral distal regions to the injury site, and contralateral uninjured regions. The relative intensity of GFAP at the proximal region over distal region (proximal/distal) was higher in PBS-treated group compared to that of sham-treated group, and was higher in WNT3A-treated group compared to that of sham-treated group (Figure 4C). These results suggest that the main effect of WNT3A is on NeuN^+^ neurons, instead of GFAP^+^ cells.

**Figure 5:**
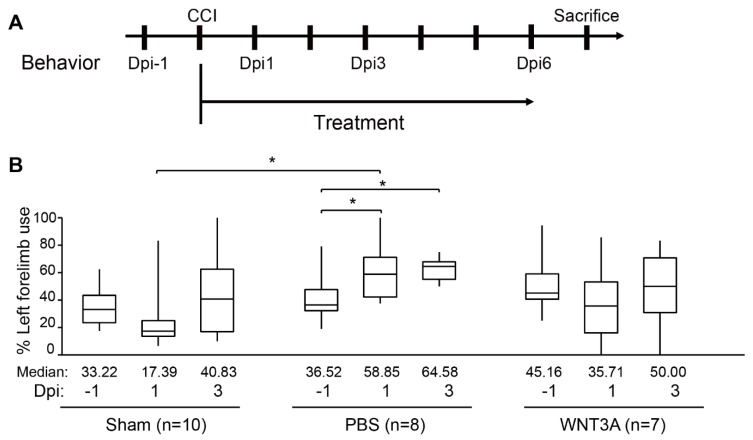
Intranasal administration of WNT3A preserves motor function of CCI mice. (**A**) Timeline of the experimental design for cylinder test. Dpi, day post injury. (**B**) The box plots of mice forelimb placing capacity in the left (ipsilateral unimpaired) forelimb before and after CCI for sham control (*n* = 10), PBS control (*n* = 8), and WNT3A treatment (*n* = 7) (−1 to 3 dpi). The boundaries of the boxes closest and farthest to the zero point indicate the 25th and the 75th percentiles, respectively. The black bars within the boxes mark the median and the values are presented below the plot. The limit of whiskers above and below the boxes mark the maximal and minimal values respectively. * *p* ≤ 0.05, analyzed by Two-way ANOVA followed by Tukey’s test.

Functional recovery of damaged motor and somatosensory brain regions was assessed by cylinder test conducted 1 day before injury (−1 dpi), 1, and 3 dpi to quantify the asymmetry use of forelimb (Figure 5A). If the left brain was injured, motor function of the right forelimb would be impaired. A preferred usage of left forelimb (asymmetry use) would be observed. As revealed in Figure 5B, sham-treated mice exhibited 10% variation of asymmetry score from 1–3 dpi compared to that of −1 dpi. PBS administration increased 20–25% asymmetry score from 1–3 dpi compared to that of −1 dpi, reflecting obvious brain injury. WNT3A administration reduced asymmetry score 10% on 1 dpi and increased 5% on 3 dpi. Together, these functional assays demonstrate that WNT3A administration preserves the motor function of mice upon traumatic brain injury.

## 3. Discussion

Upon neural injury, various signaling pathways (e.g., STAT3, BMP signaling) are triggered in reactive astrocytes, followed by the production of growth factors (e.g., BDNF, NGF), cytokines (e.g., IL-6, CNTF, CT1), and secreted proteins that are permissive to neuronal regeneration and proliferation of glial cells [17,18,19,20]. The current study characterized the positive effect of WNT3A during regeneration of injured cortical neurons, brain slices, and CCI mice model. A major challenge for treating TBI lies in the efficiency of drug delivery and cellular uptake, which is limited by the BBB. In healthy brains, the BBB restricts the influx of small molecules from circulation [21,22]. Although WNT3A is not likely to pass through the BBB in an uninjured brain due to its size, the BBB is compromised in response to TBI and may be permissive to WNT3A uptake after intravenous injection [23]. On the other hand, intranasal delivery of proteins is believed to bypass the BBB in animal models [24], and our results demonstrate that this method of WNT3A delivery can produce a promising outcome on regeneration of injured brain neurons. The clinical efficacy of systemic or intranasal administration of protein drugs to the brain of TBI patients remains to be tested [25,26]. Alternatively, small-molecule activators of WNT3A, such as 6-bromoindirubin-3′-oxime (BIO), may be readily permeable to the BBB and could be considered for clinical use. 

In conclusion, this study identified *WNT3A* as a RAG, of which expression is induced during regeneration of injured cortical neurons. Administration of WNT3A protein promotes neuronal regeneration.

## 4. Materials and Methods

### 4.1. Animals and Ethics Approval

All experiments were executed in accordance with the guidelines of the Laboratory Animal Center of National Tsing Hua University (NTHU; No. 101, Section 2, Kuang-Fu Road, Hsinchu, Taiwan), and protocols were approved by the NTHU Institutional Animal Care and Use Committee (approval# 10658, approved on 01 April 2018).

### 4.2. Reagents

Powder of Minimum Essential Medium (MEM), fetal bovine serum (FBS), horse serum (HS), penicillin-streptomycin (PS), B-27™ supplement, L-glutamine (L-Gln), Antibiotic-Antimycotic (AA), TRIzol reagent, Lipofectamine 2000, Alexa Fluor 488, 555 IgG secondary antibodies, and 4′, 6′-Diamidino-2-phenylindole (DAPI) were purchased from Invitrogen (Carlsbad, CA, USA). Neurobasal^®^ medium, Eagle’s basal medium (BME), and N-2 Supplement were purchased from Gibco (Grand Island, NY, USA). Poly-l-lysine (PLL), glutamate, Cytosine-β-d-arabinofuranoside (AraC), bovine serum albumin (BSA), sucrose, formaldehyde solution, chloroform, phenol solution, and IWR-1 were from Sigma-Aldrich (Saint Louis, MO, USA). WNT3A recombinant protein was purchased from PeproTech (Rocky Hill, NJ, USA). WNT8A and WNT9B were from R&D Systems (Minneapolis, MN, USA). *Power* SYBR Green Master Mix were purchased from Thermo Fisher Scientific (Waltham, MA, USA). Anti-Tau (sc-5587) and anti-MAP2 (sc-56561) antibodies were purchased from Santa Cruz Biotechnology (Dallas, TX, USA). Anti-TUJ1 antibodies (802001 and 801202) were purchased from BioLegend (San Diego, CA, USA). Anti-GFAP (GTX108711), anti-NeuN (GTX30773) antibodies, and rabbit IgG (GTX26702) were from GeneTex (Irvine, CA, USA) or Novus (NB300-141, Centennial, CO, Canada) for cryosection staining.

### 4.3. In Vitro Culture of Primary Neurons, Injury/Regeneration Assay, Neurite Tracking, Immunofluorescence Staining, and Quantification of Neurite re-Growth

Sprague–Dawley rats were purchased from BioLASCO Taiwan Co., Ltd. (Taipei, Taiwan) Primary cortical neurons were dissociated from dissected cortices of rat embryos (E18) and were seeded on PLL-coated plates for in vitro culture as previously described [27,28]. For the injury assay, neurons were injured on DIV8 by scraping with a p20 tip to generate injured gaps. For samples with WNT recombinant protein treatments, cortical neurons were pre-treated with WNT3A (50 ng/mL), WNT8A (100 ng/mL), WNT9B (100 ng/mL), or phosphate buffered saline (PBS) control 1 h prior to injury. For treatment with WNT inhibitor IWR-1, cortical neurons were treated with IWR-1 (10 µM) or DMSO control (1%) immediately after injury. Neurite re-growth was monitored from iDIV8 to iDIV11 in the same field-of-view by using the “Mark and Find” positioning function of AxioVision SE64 Rel. 4.9.1. software (Zeiss, White Plains, NY, USA). Live images were captured on a Carl Zeiss Observer Z1 microscope. The average width of injured gaps was calculated from measurements made at twelve positions in each gap. The percentage of gap closure was calculated as 100% × (1 − remaining gap distance/original gap distance). Up to six injured gaps were quantified per condition in at least three independent experiments.

To track re-growth of individual neurons, the tracking function of AxioVision SE64 Rel. 4.9.1. software (Zeiss, White Plains, NY, USA) was used on live images from the injury assay. From iDIV8 to iDIV11, the re-growth of 12 single neurites per field was tracked in three injured gaps in three independent experiments. The tips of regenerating neurites were located at 24-h intervals after injury, and the length of extension was measured for each day. The average length of neurite re-growth per day and the average cumulative re-growth (0–72 h) were calculated.

To better visualize neurites, neurons were fixed, blocked, and incubated with specific primary antibodies. Fluorescence images were taken using a Carl Zeiss Observer Z1 microscope.

### 4.4. Superarray qPCR and Gene Expression Analysis

For superarray analysis, total RNA was extracted from cortical neurons and used in a WNT Signaling Pathway-focused PCR Array (SuperArray Bioscience Corporation, Frederick, MD, USA). Differential gene expression levels were calculated.

For gene expression analysis, total RNA from neurons was isolated at indicated time points and processed with a High-Capacity cDNA Reverse Transcription Kit (Applied Biosystems, Waltham, MA, USA). Gene expression was analyzed by qPCR using *Power* SYBR Green Master Mix and an ABI StepOnePlus, calculated by the ddCt method, and normalized to *GAPDH*.

### 4.5. Organotypic Brain Slice Culture

Brains isolated from E18 rats were washed with HBSS medium and embedded in 2.5% low-melting agarose gel. The brain was then soaked in the artificial cerebrospinal fluid (ACSF: 125 mM NaCl, 5 mM KCl, 1.25 mM NaH_2_PO_4_, 1 mM MgSO_4_, 2 mM CaCl_2_, 25 mM NaHCO_3_, and 20 mM glucose; pH 7.4), pre-pumped with 95% O_2_/5% CO_2_, and sectioned by a Leica microtome VT100. Coronal tissue sections with a thickness of 350 µm were collected and injured with a scalpel. Sliced tissues were cultured on PLL-coated inserts in 66% BME medium supplemented with 25% HBSS, 5% FBS, 1% N-2 Supplement, 1% P/S, and 0.66% (wt/vol) D-(+)-glucose. For WNT3A treatment, WNT3A recombinant protein (50 ng/mL) was added daily, along with fresh culture medium. AraC (5 μM) was administered from DIV0 to DIV2 to limit the proliferation of glial cells.

For immunofluorescence staining of brain slices, injured brain slices were fixed with 4% PFA for 2 h at room temperature on iDIV4. The insert membrane with the attached brain slice was trimmed and tissues were subjected to immunostaining with anti-TUJ1 and anti-GFAP antibodies at dilutions of 1:750 and 1:500, respectively, for overnight incubation at 4 °C, followed by overnight incubation of 1:1000 secondary antibodies at 4 °C. DAPI was used to stain the nuclei at room temperature for 2 h. The brain slices were mounted and images were taken with a Carl Zeiss LSM780 confocal microscope. For quantification of regenerating injured brain slice, the area containing regenerating neurites and the length of injured tissue border were measured by Zeiss ZEN 2.3 lite Imaging Software (Zeiss, White Plains, NY, USA). The length of regenerating neurites was calculated as (area/length).

### 4.6. Controlled Cortical Injury (CCI) Model

Eight-to-ten-week-old C57BL/6J male mice were purchased from National Laboratory Animal Center, Taiwan, and subjected to TBI by using the pneumatic CCI equipment (Electric Cortical Contusion Impactor, Custom Design & Fabrication, Inc., Sandston, VA, USA). Upon anesthesia, craniectomy was then performed to remove the skull in a shape of 3-mm-diameter circle over the left frontoparietal cortex (−0.5 mm anteroposterior and +0.5 mm mediolateral to bregma). After exposing the dura matter, the brain was impacted by pneumatically operated CCI device at the velocity of 3 m/s, reaching 1 mm in depth, affecting a 2-mm-diameter circular area, and with 500-ms dwell time to generate mild TBI [13]. After CCI-induced brain injury and suturing, PBS or 50 ng WNT3A was intranasally delivered to mice daily for consecutively four days. On 4 dpi, the TBI mice were anesthetized and perfused with saline and 4% paraformaldehyde. The brain tissues were then isolated, immersed in 10%, 15%, and 20% sucrose followed by embedded in Shandon Cryomatrix embedding resin (Thermo Scientific, Waltham, MA, USA) and sliced into 10-μm sections by cryostat microtome (Leica CM3050 S, Leica Biosystems, Baffalo Grove, IL, USA). The cryosections were incubated in antigen retrieval solution at 70 °C for 20 min and then incubated in 1% BSA for 2 h. After BSA blocking, the cryosections were subjected to immunofluorescence staining with anti-TUJ1 and anti-GFAP antibodies, followed by incubating with fluorescence-conjugated secondary antibodies. DAPI was used to stain the nuclei at room temperature for 10 min. Finally, the cryosections were mounted by using 90% glycerol. Fluorescence images of brain cryosections were imaged by Zeiss Confocal LSM800 and analyzed by Zeiss ZEN 2.3 lite Imaging Software (Zeiss, White Plains, NY, USA). The intensity of GFAP around the impact site (indicated as “proximal”), at the ipsilateral hemisphere distal to the impact site (indicated as “distal”), and at the contralateral uninjured hemisphere (indicated as “uninjured”), was quantified separately by calculating the average GFAP intensity of 4 defined squared region of interest (ROI) in each brain region. Relative intensity of GFAP was defined as the average intensity of proximal regions divided by the average intensity of distal or uninjured regions (Figure 4C, left panel). The number of NeuN^+^ cells was defined as the average NeuN^+^ cells counted in the 4 squared ROI at the proximal regions around the impact site. The area of each ROI is 0.05 mm^2^.

### 4.7. Behavior Assays for Functional Recovery

Asymmetry use of forelimb was measured to represent functional recovery of damaged brain region. To this end, the cylinder test was conducted 1 day before injury (−1 dpi, day post injury), and 1–3 dpi, at 7–9 pm to quantify the asymmetry use of forelimbs. The protocol of cylinder test was modified according to Hua, Y et al.’s [29]. Briefly, C57BL/6 mice were put in a transparent cylinder of plexiglass and the behavior was recorded. The number of times that the forelimbs contact on the cylinder wall was calculated in 5 min. Valid forelimb–wall contacts were considered for further data quantification if the forelimbs were raised over the shoulder of the mice. Data of cylinder test were quantified by calculating the percentage of the unimpaired left forelimb use (ipsilateral limb to the CCI site), which was calculated by following parameter: (1) The frequency of the mice using its left forelimb first (U, unimpaired), (2) the frequency of the mice using its right forelimb first (I, impaired), and (3) the frequency of the mice using both forelimbs (B, both). The percentage of the unimpaired limb use was calculated as [U/(U + I + B)], indicated as “% left forelimb use”. To minimize bias, data collected from behavior experiments of sham group animals demonstrating slight brain injury or CCI animals failed to be injured at expected brain regions were excluded, according to immunostaining of the cryosections. Among available behavior recordings, only data where forelimb contact equals to or more than three times in the given duration were included.

### 4.8. Statistical Anaylsis

All results are expressed as mean ± SEM or box-and-whisker plot from at least three independent experiments unless otherwise noted. Paired Student’s *t*-test was performed using Microsoft^®^ Excel^®^ 2016 MSO (16.0.4849.1000) 64 bits for Figure 2B and Figure 3A. Prism v.8.0.2 (GraphPad Software, available online: https://www.graphpad.com/scientific-software/prism/) was used to perform non-parametric Mann–Whitney test (Figure 3E), one-way ANOVA followed by Tukey’s test (Figure 4B,C), and two-way ANOVA followed by Tukey’s test (Figure 5B). Statistical significance (*) is defined as *p* ≤ 0.05.

## Abbreviations

TBI	Traumatic brain injury
CNS	Central nervous system
RAG	Regeneration-associated gene
DIV	Day in vitro
iDIV	Injured day *in vitro*
AraC	Cytosine-β-D-arabinofuranoside
MAP2	Microtubule-associated protein 2
BBB	Blood-brain barrier
CCI	Controlled cortical injury
Dpi	Day post injury
GFAP	Glial fibrillary acidic protein
